# Effect of atmospheric pressure plasma jet on the structure and physicochemical properties of wheat starch

**DOI:** 10.3389/fnut.2024.1386778

**Published:** 2024-05-03

**Authors:** Hongfang Ji, Dandan Li, Lingwen Zhang, Manjie Li, Hanjun Ma

**Affiliations:** ^1^School of Food Science, Henan Institute of Science and Technology, Xinxiang, China; ^2^National Pork Processing Technology Research and Development Professional Center, Xinxiang, China

**Keywords:** atmospheric pressure plasma jet, discharge power, wheat starch, physicochemical properties, structure

## Abstract

The effect of atmospheric pressure plasma jet (APPJ) with different discharge power (0, 400, 600, and 800 W) on the structure and physicochemical properties of wheat starch were evaluated in this study. After APPJ treatments, significant declines in peak viscosity, breakdown viscosity, and final viscosity of wheat starch pasting parameters were observed with increase of plasma treatment power. Being treated with discharge power of 800 W, the PV and BD value of wheat starch paste significantly dropped to 2,578 and 331 cP, respectively. Apparently, APPJ could raise the solubility of wheat starch, while reduce the swelling capacity, and also lower the G′ and G″ value of wheat starch gel. Roughness and apparent scratch was observed on the surface of the treated wheat starch granules. Although APPJ treatment did not alter wheat starch’s crystallization type, it abated the relative crystallinity. APPJ treatment might be useful in producing modified wheat starch with lower viscosity and higher solubility.

## 1 Introduction

Wheat starch is the main component of wheat (*Triticuma estivum* L.), and accounts for 65–75% of wheat grains (based on the dry weight) (1). Wheat starch can be used in all kinds of foods such as steamed bread, deep-fried dough sticks, pasta, noodles, bread, cakes, etc., and has higher commercial value (2). Although natural wheat starch has the advantages of rich source and low cost, its application in food industry is limited because of its low water solubility, prone to aging and retrogradation (3). To overcome the upper disadvantages, the chemical, physical, and enzymatic methods are usually employed to modify starch (4–8). In recent years, the non-thermal physical modification methods for starch has been received much attention for environmental protection, higher efficiency, and easy operation (9).

Cold plasma is partially or completely ionized gas, which could be produced by using dielectric barrier discharge (DBD), glow discharge, radio frequency discharge, resistive barrier discharge, microwave discharges, corona discharge, and atmospheric pressure plasma jet (APPJ) to neutral gas with atmospheric or low pressure (10). Cold plasma contains several reactive substances, including singlet oxygen, ozone, hydroxyl radical, nitric oxide, hydrogen peroxide, superoxide anion, nitrite, etc. (11). As an emerging green physical modification method, cold plasma has shown great application in starch modification due to its advantages such as low energy consumption, higher efficiency, and fewer by-products (12). Ranjitha et al. (13) reported that, being treated by pin to plate cold plasma under the ordinary pressure, the surface of mango kernel starch granules was etched, its starch chain was depolymerized. Modified by radio frequency air plasma, the surface of cassava starch granules was also etched, resulting in the increased hydrophilicity (14). The viscosity of corn starch increased after being modified by DBD at 50 V for 5 min (15). In another study, glow-plasma (the discharge gas was nitrogen and helium) caused the viscosity parameters of potato starch paste decline (16). Additionally, ethylene glow discharge plasma could significantly reduce the decomposition temperature of starch (17). It could be observed that cold plasma could obviously affect not only the structure feature but also the functional properties of starch.

Many scholars have carried out systematic research on starch modification by DBD discharge, but few have reported on the effect of APPJ discharge on starch modification, especially on the structure and physicochemical properties of wheat starch. In this study, wheat starch was treated by APPJ with different discharge power, the changes of physicochemical properties such as solubility, swelling capacity, rheological, and pasting parameters were investigated. The effect of APPJ treatment on structural features (amylose content, crystal structure, and morphology of starch) was also analyzed.

## 2 Materials and methods

### 2.1 Materials and chemicals

Wheat flour (it is prepared with wheat of Bainong 4199 variety, which is widely cultivated in Henan Province, China) was purchased from Jiyuan Xuemiao Wheat Deep Processing Technology Company, Jiyuan, China. Wheat starch was prepared from wheat flour according to the method in the literature reported by Punia et al. (18). All the other chemical reagents (such as potassium iodide, ethanol, potassium bromate, sodium hydroxide, hydrochloric acid, etc.) utilized in this paper were of analytical grade.

### 2.2 Atmospheric pressure plasma jet modification of wheat starch

**Figure 1:**
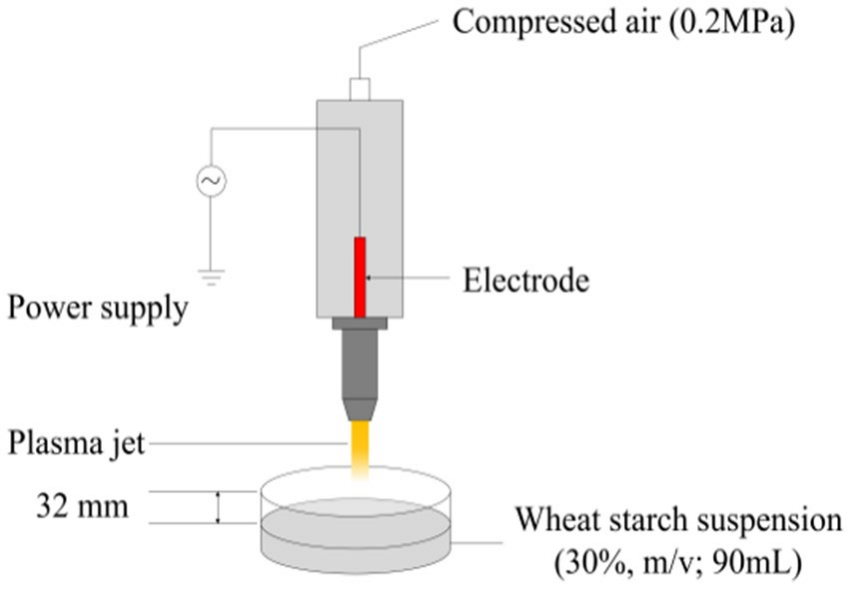
The schematic diagram of APPJ device.

The schematic diagram of APPJ employed in present research was shown in Figure 1. 90 mL of wheat starch suspension (30%, m/v) was modified by APPJ apparatus (Henan Xiantu Zhineng Co., Ltd., Zhengzhou, China). Compressed air under pressure of 0.2 MPa was used as the working gas, and the velocity of flow was set at 50 L/min. The distance from the surface of wheat starch slurry to the plasma jet nozzle was 32 mm. The starch samples were treated by APPJ at three different input discharge powers (0, 400, 600, and 800 W) for 3 min. After being washed with double distilled water, the APPJ treated wheat starch samples were dried for 60 h at 40°C.

### 2.3 Solubility and swelling power measurement

The solubility and swelling capacity of all wheat starch samples were examined following the method described by Liu and Shen (19) with slight modification. Suspension of wheat starch (1%, w/v) was kept at 55–85°C for 0.5 h with intermittent stirring. After being cooled down to ambient temperature (25°C), the mixture was centrifuged for 25 min at 7,000 × *g*. The supernatant was collected, and then dried for 4 h at 110°C. The weight of starch sample (m), the dissolved solids in the supernatant (m_1_), and the dissolved solids in sediment (m_2_) was obtained. The swelling power and solubility was calculated as follows:


$$\begin{array}{c} \mathrm{Solubility}\;\left(\%\right)=\left[m_{1}/m\right]\times 100;\mathrm{Swelling power}\\ =\left[m_{2}/\left(m\;\left(1-\mathrm{Solubility}\right)\right)\right]\times 100 \end{array}$$

### 2.4 Pasting property

The RVA-4500 visco analyzer (Hagersten, Sweden) was utilized to examine the pasting parameters of native-WS and APPJ treated wheat starch. The measurement conditions were set up according to the published method (20). 2.5 g of wheat starch samples was mixed with 25 mL of distilled water. The test conditions were set as follows: samples were equilibrated at 50°C for 1 min, heated to 95°C at the rate of 12°C/min, kept at 95°C for 2.5 min, then cooled to 50°C at 12°C/min, and held for 2 min. The parameters of starch pasting were determined.

### 2.5 Rheological properties

The Haake Mars 60 rheometer was employed to measure the dynamic rheological parameters of wheat starch samples. Frequency sweep test parameters were set according to the method described by Ji et al. (21). The values of G″, G′, and tan δ (G″/G′) were obtained.

### 2.6 Amylose content examination

The amylose amount of native-WS and APPJ treated wheat starch was examined according to the reported method of Guo et al. (22). 10 mg of wheat starch samples was dispersed in 8 mL 90% dimethyl sulfoxide and stirred constantly in a boiling water bath for 20 min. After being cooled to room temperature, 5 mL of iodine solution (2.5 mM I_2_/6.5 mM KI) was added and mixed completely. Subsequently, the mixture was diluted to 50 mL with distilled water and mixed vigorously. The absorbance was detected at 600 nm and amylose content was calculated.

### 2.7 X-ray diffraction

The XRD patterns of wheat starch were obtained by D8 Advance Bruker XRD (Germany), measured as described by Sun et al. (20). The test conditions were as follows: radiation source was Cu-Kα at a voltage of 40 kV and a current of 100 mA, the 2θ angle was set in the range from 4 to 40°, and the scanning speed was with 2°/min. And then the degree of relative crystallinity was calculated.

### 2.8 FT-IR spectroscopy

According to the published method (23), a Tensor 27 FT-IR spectroscopy (Billerica) was used to analyze wheat starch samples. Wheat samples were mixed with KBr (the ratio of sample to KBr was 1:100, w/w). And then, the mixture was pressed into filmy pellet. The scanning wavelength range was set from 4,000 to 500 cm^−1^, and the times of average scan time were 16. The scanning was carried out with the speed of 4 cm^−1^.

### 2.9 Morphological characterization observation

The morphological characteristics of wheat starch samples were observed using a FEI Quanta 200 SEM (United States). After being gold-plate, the native-WS and APPJ treated wheat starch was observed at voltage of 15 kV, and the representative images were recorded at the magnification of 2,000×.

### 2.10 Statistical analysis

Results were displayed as the mean ± standard deviation (SD). Origin 2016 was used to draw the graphs. The statistical comparison was based on LSD test by using SPSS26.0, and the significance was set at *p* < 0.05. All experiments were carried out in triplicates.

## 3 Results and discussion

### 3.1 Solubility and swelling power

The solubility of APPJ treated wheat starch was much higher than that of native-WS, and with the increase of APPJ discharge power, the solubility of modified wheat starch noticeably increased (Table 1). Similar results had been previously observed (24, 25). The reason for the increase of starch solubility was that active species in plasma could partially decompose or depolymerize starch granules into smaller starch fragments, which increased the solubility (26, 27). At 55°C, the solubility of wheat starch increased from 3.97% (native-WS) to 5.62% (APPJ-P800 sample), which showed that cold plasma raised the solubility of wheat starch in non-pasting state (25). This also indicated that APPJ treatment could lose the surface structure of starch granule. At 85°C, the solubility of wheat starch significantly rose from 8.90% (native-WS) to 10.72% (APPJ-P800 sample), which indicated that APPJ treatment could improve the solubility of wheat starch in the pasting period.

**Table 1:** Solubility of native-WS and APPJ treated wheat starch samples.

Samples	Solubility (%)			
	55°C	65°C	75°C	85°C
Native-WS	3.97 ± 0.11^d^	5.48 ± 0.02^c^	6.57 ± 0.03^d^	8.90 ± 0.27^d^
APPJ-P400	4.30 ± 0.07^c^	5.99 ± 0.10^b^	7.11 ± 0.04^c^	9.47 ± 0.06^c^
APPJ-P600	4.78 ± 0.14^b^	6.08 ± 0.05^b^	7.86 ± 0.03^b^	10.01 ± 0.03^b^
APPJ-P800	5.62 ± 0.14^a^	6.26 ± 0.02^a^	8.58 ± 0.11^a^	10.72 ± 0.07^a^

Different letters between data in the same column indicate a significant difference (*p* < 0.05).

Swelling power of APPJ treated wheat starch was significantly lower than that of native starch (*p* < 0.05), and decreased with the increase of APPJ discharge power (Table 2). At 85°C, the swelling degree of wheat starch notably decreased from 15.52% (native-WS) to 10.61% (APPJ-P800 sample). This might be due to the starch chains’ destruction caused by APPJ, which led to the decline in the holding water ability of the swollen starch granules (28). Some studies had reported similar trends of decrease in swelling power such as in banana starch (25), and in maize starch (29). While, Wu et al. (24) reported that no apparent changes had been found between corn starch without modification and the samples treated with APPJ. On the contrary, the report had described that pin-to-plate cold plasma with the input voltage changing from 190 to 230 V could enhance the swelling volume of arrowroot starch (30).

**Table 2:** Swelling power of native-WS and APPJ treated wheat starch samples.

Samples	Swelling power (%)			
	55°C	65°C	75°C	85°C
Native-WS	6.70 ± 0.07^a^	9.52 ± 0.04^a^	13.67 ± 0.03^a^	15.52 ± 0.06^a^
APPJ-P400	6.38 ± 0.04^b^	9.01 ± 0.03^b^	12.24 ± 0.08^b^	13.10 ± 0.07^b^
APPJ-P600	6.23 ± 0.03^c^	8.68 ± 0.17^c^	11.65 ± 0.02^c^	12.78 ± 0.03^c^
APPJ-P800	6.13 ± 0.05^d^	8.51 ± 0.14^c^	9.33 ± 0.06^d^	10.61 ± 0.04^d^

Different letters between data in the same column indicate a significant difference (*p* < 0.05).

### 3.2 Pasting properties

Pasting parameters of the native-WS and APPJ treated wheat starch were shown in Table 3. The peak viscosity (PV), final viscosity (FV), setback viscosity (SB), and breakdown viscosity (BD) of wheat starch decreased after APPJ treatment by comparison with those of native-WS (Table 3). With the increase of plasma power, all viscosity parameters of APPJ treated wheat starch decreased. When the treatment power was 400 W, the PV, TV, BD, and FV of wheat starch was 3,458, 2,990, 469, and 4,220 cP, respectively, which was notably lower than those in native-WS. Gao et al. (28) had investigated the influence of DBD on starch’ digestion and physicochemical properties, and reported the similar results. Additionally, similar results were also found in the previous reports (16, 27). The viscosity parameters reduced when potato starch and red adzuki bean starch was modified by glow-plasma and DBD plasma, respectively (16, 27).

**Table 3:** Pasting properties of native-WS and APPJ treated wheat starch samples.

Samples	PV (cP)	TV (cP)	BD (cP)	FV (cP)	SB (cP)	GT (°C)
Native-WS	3,667 ± 62.6^a^	3,083 ± 52.6^a^	584 ± 10.0^a^	4,350 ± 74.2^a^	1,267 ± 21.5^a^	87.40 ± 0.15^a^
APPJ-P400	3,458 ± 55.9^b^	2,990 ± 48.8^b^	469 ± 7.6^b^	4,220 ± 68.1^b^	1,230 ± 19.8^a^	85.78 ± 0.79^bc^
APPJ-P600	2,916 ± 67.1^c^	2,493 ± 57.1^c^	423 ± 10.0^c^	3,549 ± 81.6^c^	1,055 ± 24.0^b^	84.58 ± 0.92^c^
APPJ-P800	2,578 ± 35.4^d^	2,247 ± 30.3^d^	331 ± 4.6^d^	3,280 ± 45.0^d^	1,033 ± 14.2^b^	86.20 ± 0.41^ab^

Different letters between data in the same column indicate a significant difference (*p* < 0.05).

The PV represented the starch granule swelling extent during its pasting period (31). The PV value of APPJ-P800 sample plunged to 2,578 cP, reduced by 29.70% compared with that of native-WS (the value was 3,667 cP), which might be accounted for the depolymerization of starch chains caused by APPJ treatment. With APPJ discharge power at 800 W, the BD value notably decreased by −43.32%, dropping from 584 cP (native-WS) to 331 cP (APPJ-P800), suggesting cold plasma could enhance the thermal and shear stability of starch samples. The SB value reflected aging property of starch after being gelatinized and cooled. The SB of APPJ treated wheat starch declined along with increase of APPJ discharge power. Remarkable decline in SB value from 1,267 cP (native-WS) to 1,033 cP (−18.47% for APPJ-P800 sample) was noted. These indicated that APPJ could enhance the stability of cooled paste of starch. Additionally, the gelatinization temperature (GT) of APPJ modified wheat starch was less than that of native-WS (the value was 87.40°C), and reached to the minimum of 84.58°C (APPJ-P600 sample), which suggested that the APPJ treatment promoted starch granules to absorb water to swell and to crack during gelatinization period.

### 3.3 Rheological properties

**Figure 2:**
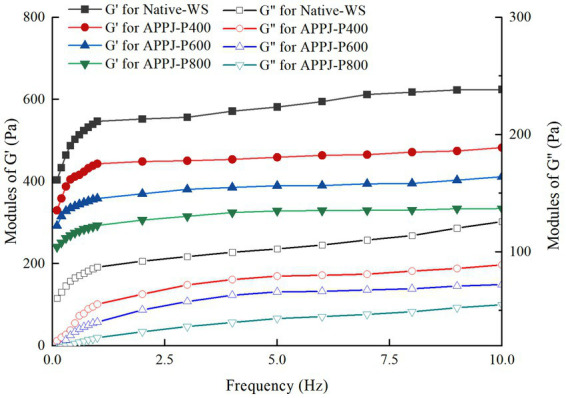
Modules of G′ and G″ for Native-WS and APPJ treated wheat starch samples with frequency.

**Figure 3:**
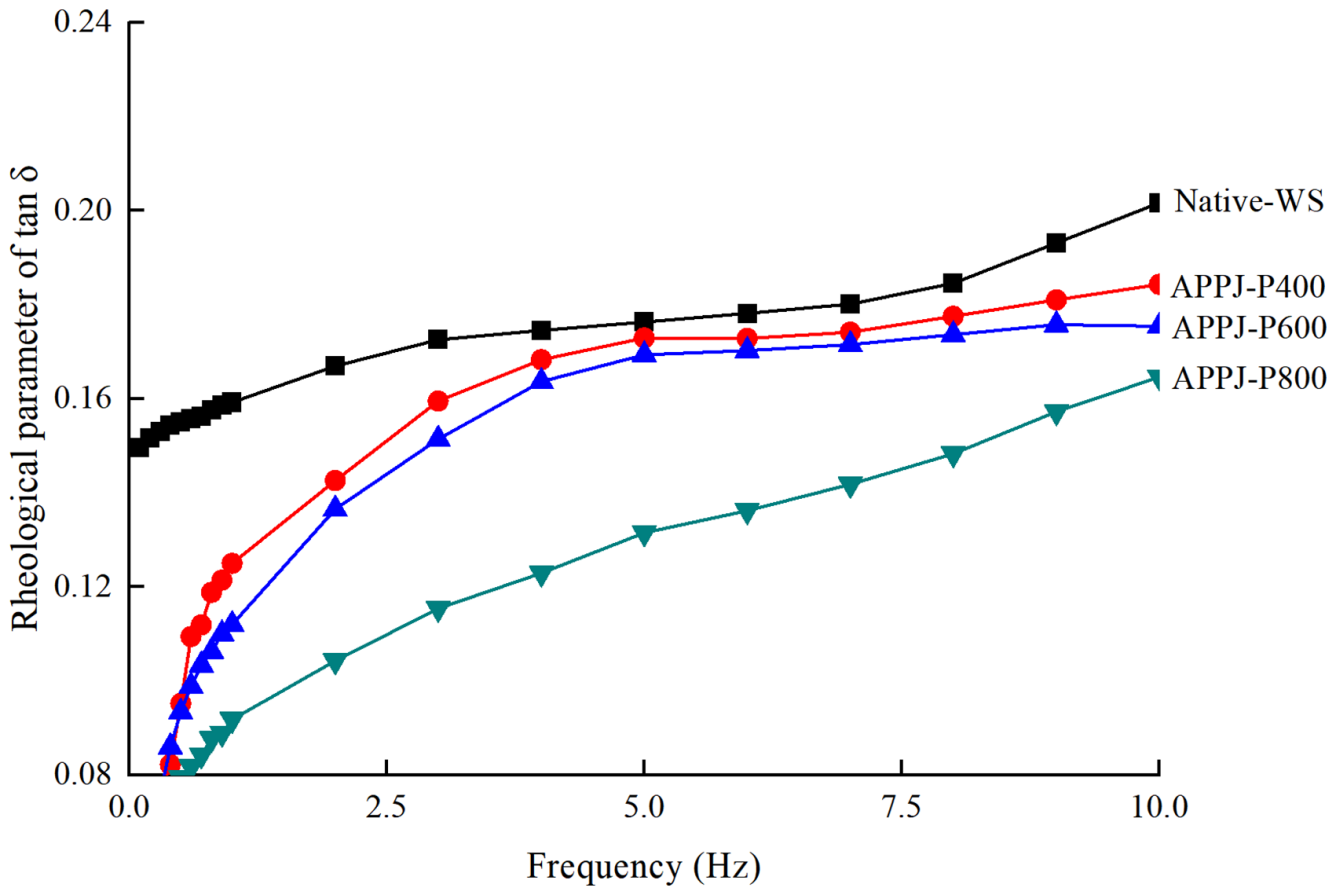
Rheological parameter of tan δ for Native-WS and APPJ treated wheat starch samples with frequency.

The rheological properties of native-WS and APPJ treated wheat starches were presented in Figures 2, 3. Figure 2 displayed that both G′ and G″ of native-WS and APPJ treated wheat starch increased with increasing frequency, which indicated that the modulus of wheat starch gel was frequency dependent. Additionally, in course of frequency variation, the G′ value of all starch samples gel was always larger than G″ value (Figure 2), and its tan δ was always less than 1 (Figure 3), which indicated that all the gels of native-WS and APPJ treated wheat starch were typical weak gel systems.

G′ of APPJ treated wheat starch gel was lower than those of the native in the measured frequency range, and had been declining with the increase of APPJ discharge power (Figure 2). These indicated that the starch gel became weaker, and was prone to be out of shape (32). It might be attributed to the molecular depolymerization of starch treated with APPJ. Additionally, APPJ treatment could also make G″ of wheat starch gel fall (Figure 2). Similar result was found by Yan et al. (25), who reported APPJ treatment lowered the G′ and G″ of corn starch gel.

The tan δ values of wheat starch gels treated with APPJ were obviously lower than those of native-WS samples (Figure 3), which revealed that the viscosity of the APPJ treated starch gel reduced quickly than elasticity, resulting in the starch gel with stronger solid-like behavior. Similar result was described in a previous study (25).

### 3.4 Amylose content

**Figure 4:**
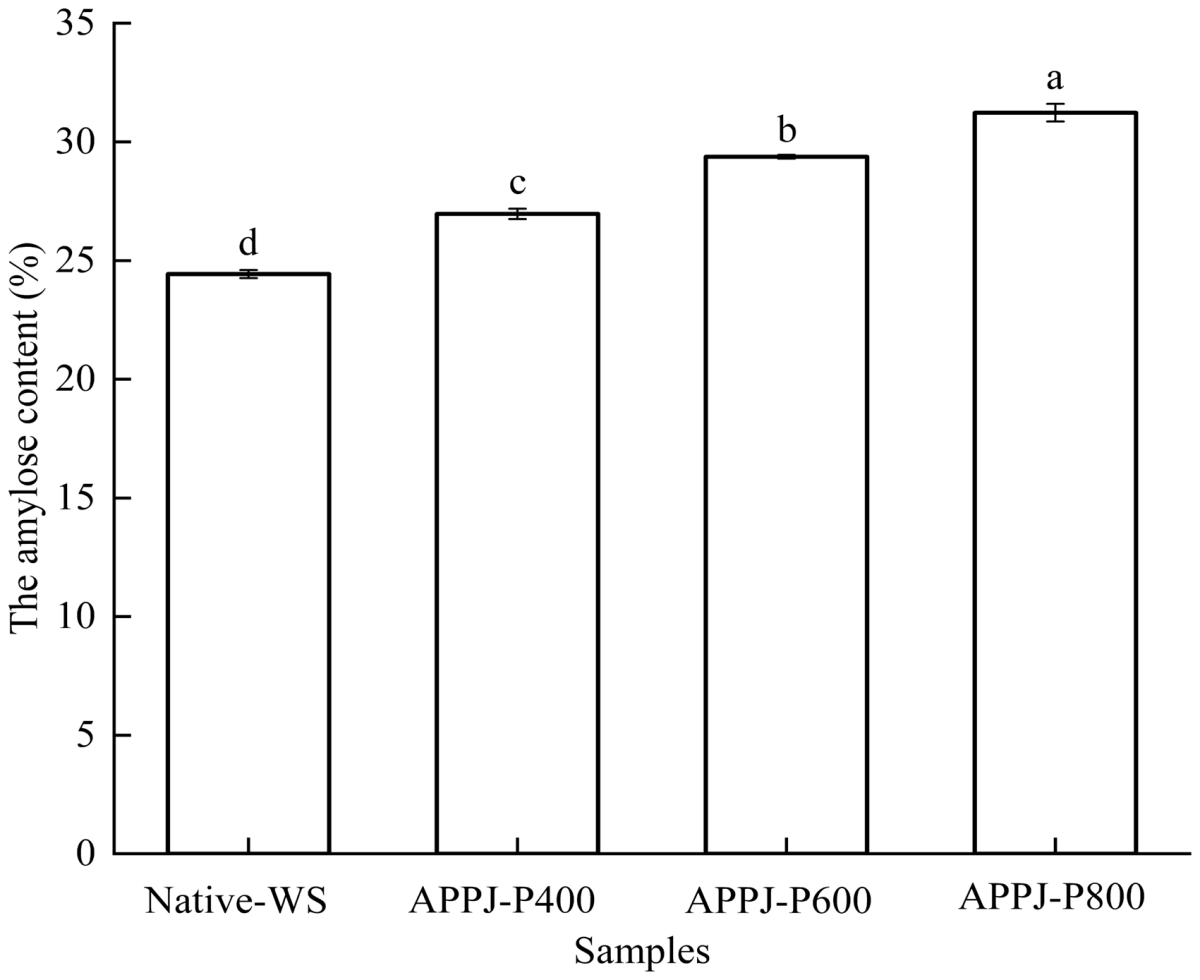
The content of amylose in native-WS and APPJ treated wheat starch samples. Different letters above the bar indicate the significant difference (*p* < 0.05).

The content of amylose in native-WS and APPJ treated wheat starch was illustrated in Figure 4. The amylose content of wheat starch is usually in range of 20–30/100 g (33). The amylose content of native-WS was 24.44/100 g, which was close to the data reported by Liu et al. (33), but lower than that found with Shen et al. (31). This difference might be due to wheat varieties, wheat planting environment, and wheat starch preparation methods.

Atmospheric pressure plasma jet treatments increased the amylose content of wheat starch (Figure 4). Treated with the higher discharge power of 800 W, the amylose content of wheat starch reached 31.23%, which increased by 27.78% than that of native-WS. Similar results were reported by Yan et al. (25). However, when corn starches were modified by cold plasma, its amylose content declined, which was observed by Banura et al. (14). These results indicated that cold plasma modification had different influence on the content of amylose in starch, which was due to starch from various sources and plasma produced by different gas component. The depolymerization of amylose chains caused by reactive species in cold plasma could be accounted for the reduce of amylose content (31). While, the higher amylose content of starch was attributed to the cleavage of α-1,6-glycosidic bonds induced by cold plasma (34, 35).

### 3.5 FT-IR analysis

**Figure 5:**
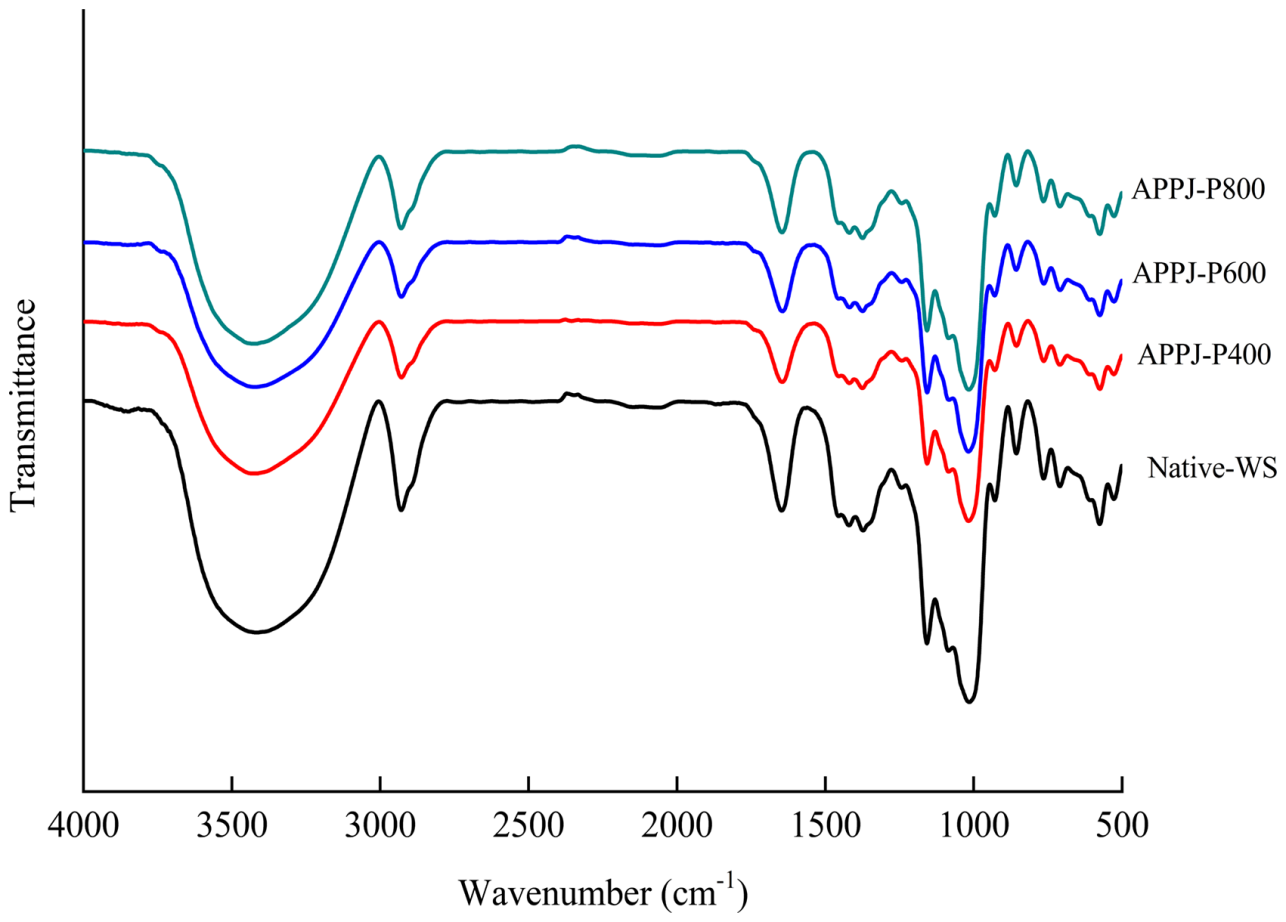
Fourier infrared spectrum of native-WS and APPJ treated wheat starch samples.

The FT-IR spectra curve of native-WS and APPJ treated wheat starch were presented in Figure 5. Native wheat starch was observed with remarkable energy bands at the fingerprint region. No new absorption peaks could be seen in FT-IR spectra (in the range from 4,000 to 500 cm^−1^) of all APPJ treated wheat starch samples (Figure 5), which displayed that APPJ could not change the chemical group of wheat starch.

**Figure 6:**
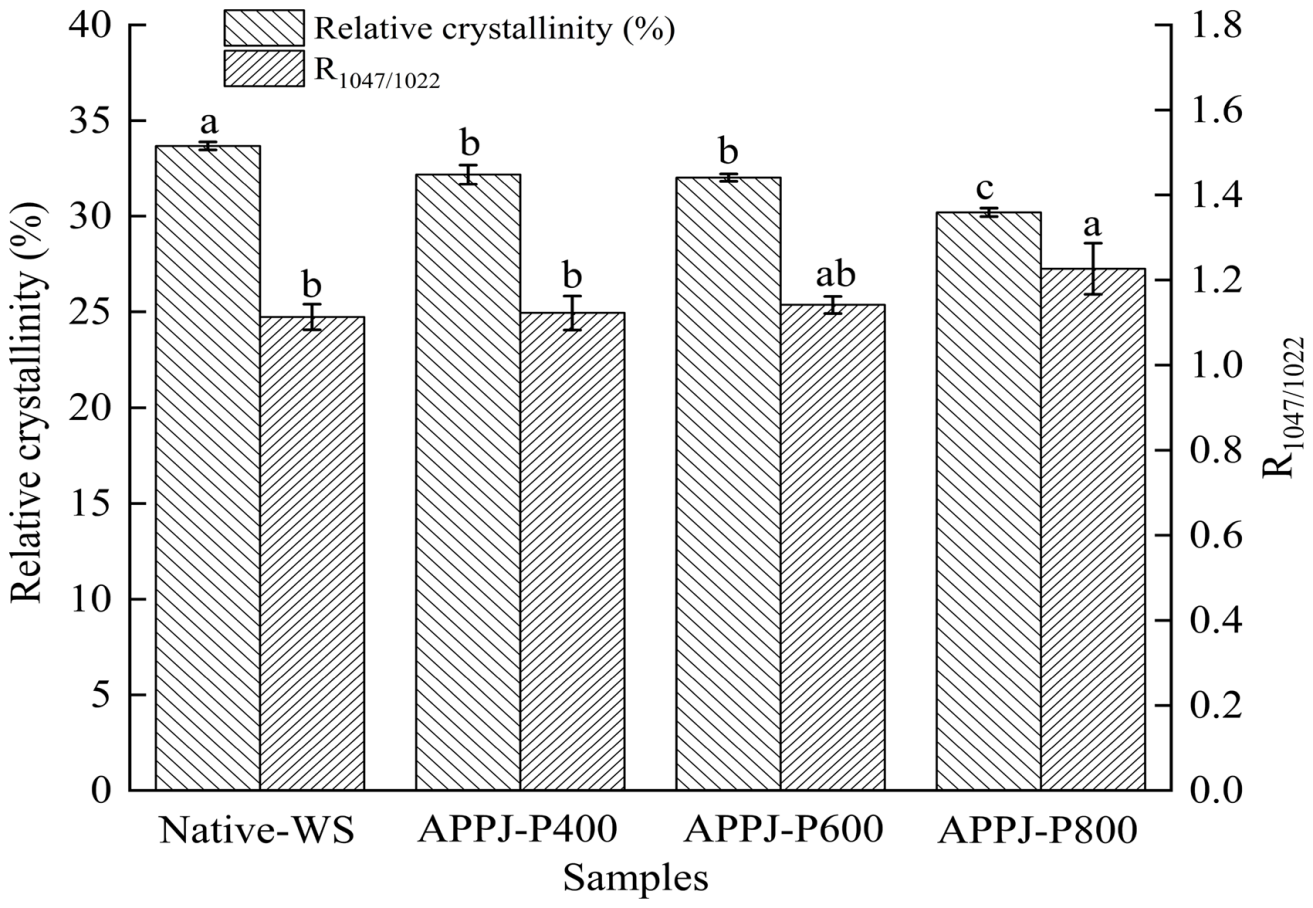
The relative crystallinity and R_1047/1022_ of native-WS and APPJ treated wheat starch. Different letters above the bar indicate the significant difference (*p* < 0.05).

As shown in Figure 6, the absorbance intensity ratio (R) of 1047/1022 value of wheat starch samples showed a rising trend along with the enhancement of APPJ discharge power. At lower discharge power (400 and 600 W), a slightly increase (*p* > 0.05) of R_1047/1022_ up to 1.12 and 1.14, respectively, were observed. At higher discharge power (800 W), R_1047/1022_ reached to highest with the value of 1.27, which was 1.1-fold higher (*p* < 0.05) than that of native-WS (the value was 1.11). These results indicated that cold plasma treatment facilitated the formation of the ordered short-range double helices at the starch granule surface layers. Similar results had been demonstrated in the previous reports of Sun et al. (20), and Yan et al. (25), in which both banana and rice starch treated with DBD plasma significantly increased the value R_1047/1022_ in starch granule. As could be seen in Figure 6, the change tendency in R_1047/1022_ of APPJ treated wheat starch was inconsistent with the variation of relative crystallinity analyzed by XRD. The reason for the above results might be that FT-IR could only penetrate the starch granule surface. While, the X-ray could penetrate the whole starch granules (3).

### 3.6 Crystallization characteristics

**Figure 7:**
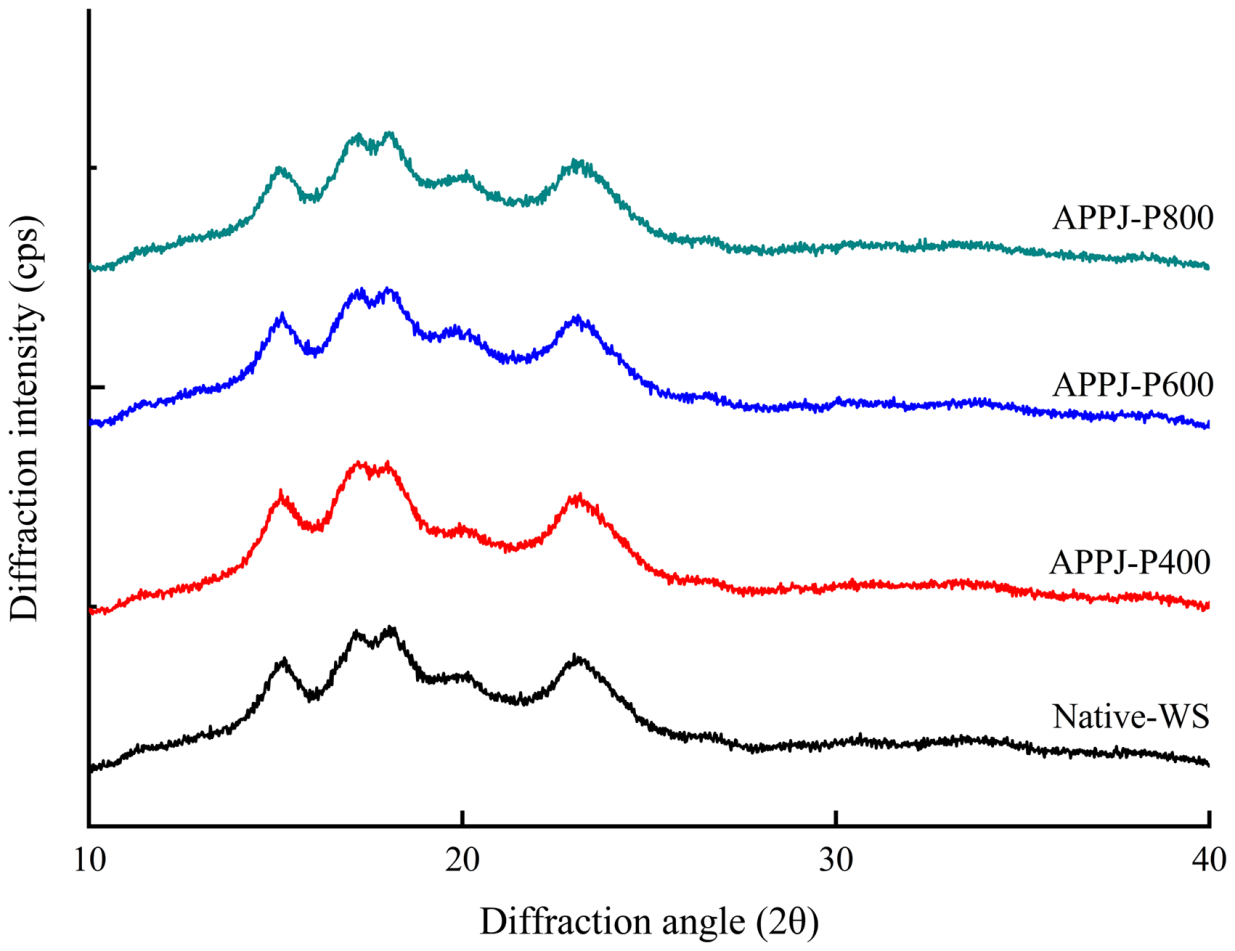
XRD spectra of native-WS and APPJ treated wheat starch.

Starch granule is a typical semi-crystalline crystal composed of crystalline region and amorphous zone. The XRD patterns of native-WS and APPJ treated wheat starch samples were illustrated in Figure 7. The native-WS and all APPJ treated wheat starch samples showed A-type crystalline structure, and the diffraction angles with definite sharp peaks were observed at 15, 17, 18, 20, and 23^o^ (Figure 7). This indicated that APPJ treatment did not change the crystalline polymorph of wheat starch. These results were consistent with the previous studies (36).

Being compared with the relative crystallinity of native-WS, the value of wheat starch modified by APPJ with different discharge power obviously declined (Figure 6). The relative crystallinity became lower with the increase of APPJ treatment power, and decreased from 33.68% (the native-WS) to 30.20% (800 W APPJ treated wheat starch). After being modified by glow discharge plasma for 10 min at 40 W, the relative crystallinity of rice starch reduced to the value of 37.47%, notably lower than that of the native starch sample (43.06%), which was found by Thirumdas et al. (37). The decrease of relative crystallinity could be accounted for the starch molecules depolymerization bombarded by reactive species in plasma (32). Additionally, the interaction between reactive species in plasma and chains of starch molecules might induce the molecular scission and granular corrosion of starch, which resulted in the decrease in crystallinity (38). The stronger the cold plasma discharge power or the longer the cold plasma action time, the higher depolymerization rate of starch, which resulted in the lower relative crystallinity of starch (25, 31).

### 3.7 Morphology of granules

**Figure 8:**
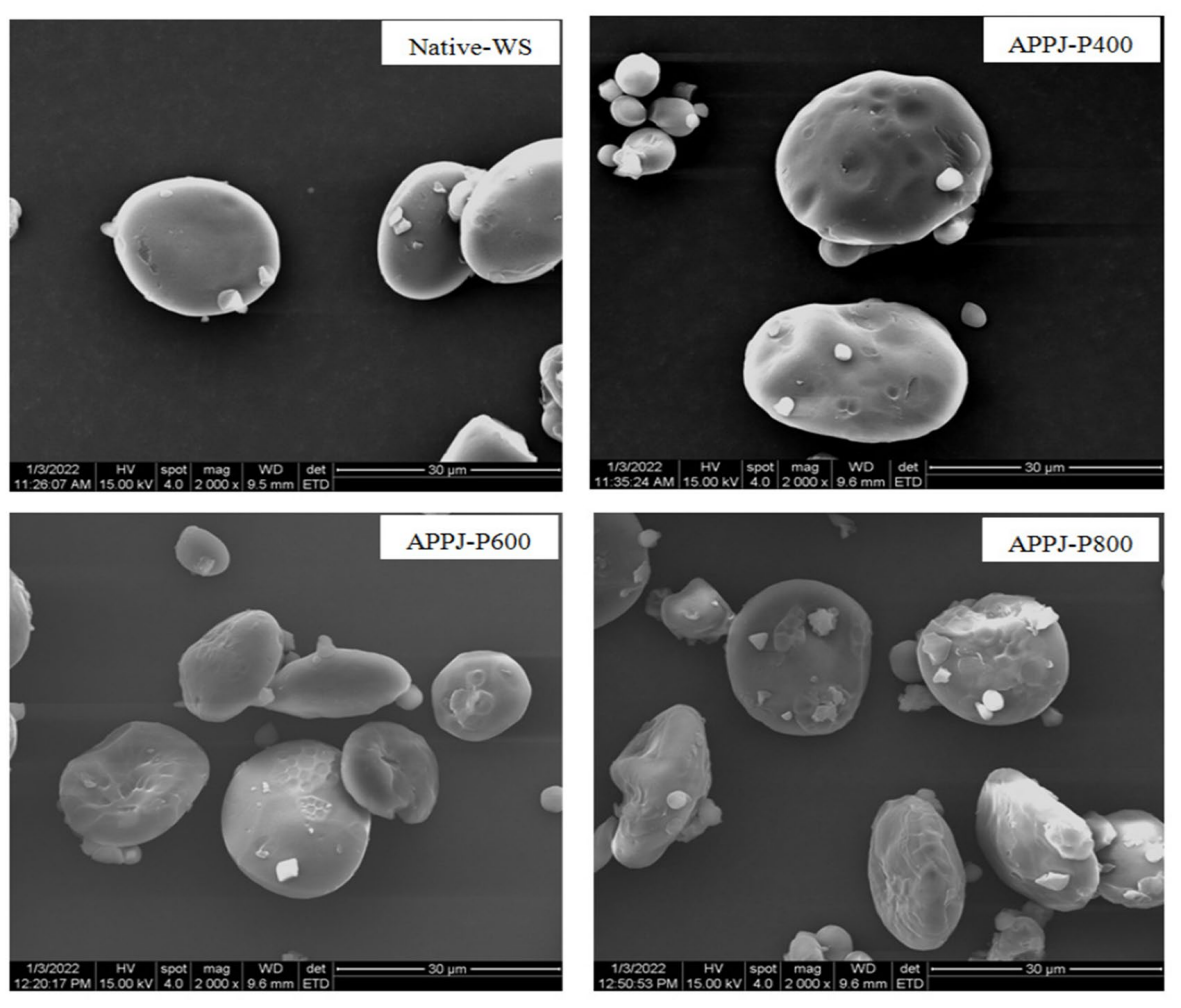
Scanning electron microscopy of native-WS and APPJ treated wheat starch samples (2,000×).

The SEM microstructure images of native-WS and APPJ treated wheat starch were illustrated in Figure 8. The surface of native wheat starch granules was smooth, almost without fissures or scratches. After APPJ treatments, no significant changes in the overall morphology of all starch samples were observed. While, surface roughness, dents, and apparent scratch was observed on the surface of wheat starch granules modified by APPJ with different discharge power. Previous works had also demonstrated the similar results (27, 39, 40). Fissures or cavities were found on the surface of starch granules, which was observed by Sudheesh et al. (39). Additionally, the higher the cold plasma treatment power, the more obvious the surface etching, similar result was described by Thirumdas et al. (41). In another study, it was interesting that nitrogen plasma had no effect on starch granules while helium plasma caused surface corrosion of starch (16). These differences might be attributed to the variance of plasma active components produced by various gas.

### 3.8 The potential mechanism of the effect of APPJ on wheat starch

**Figure 9:**
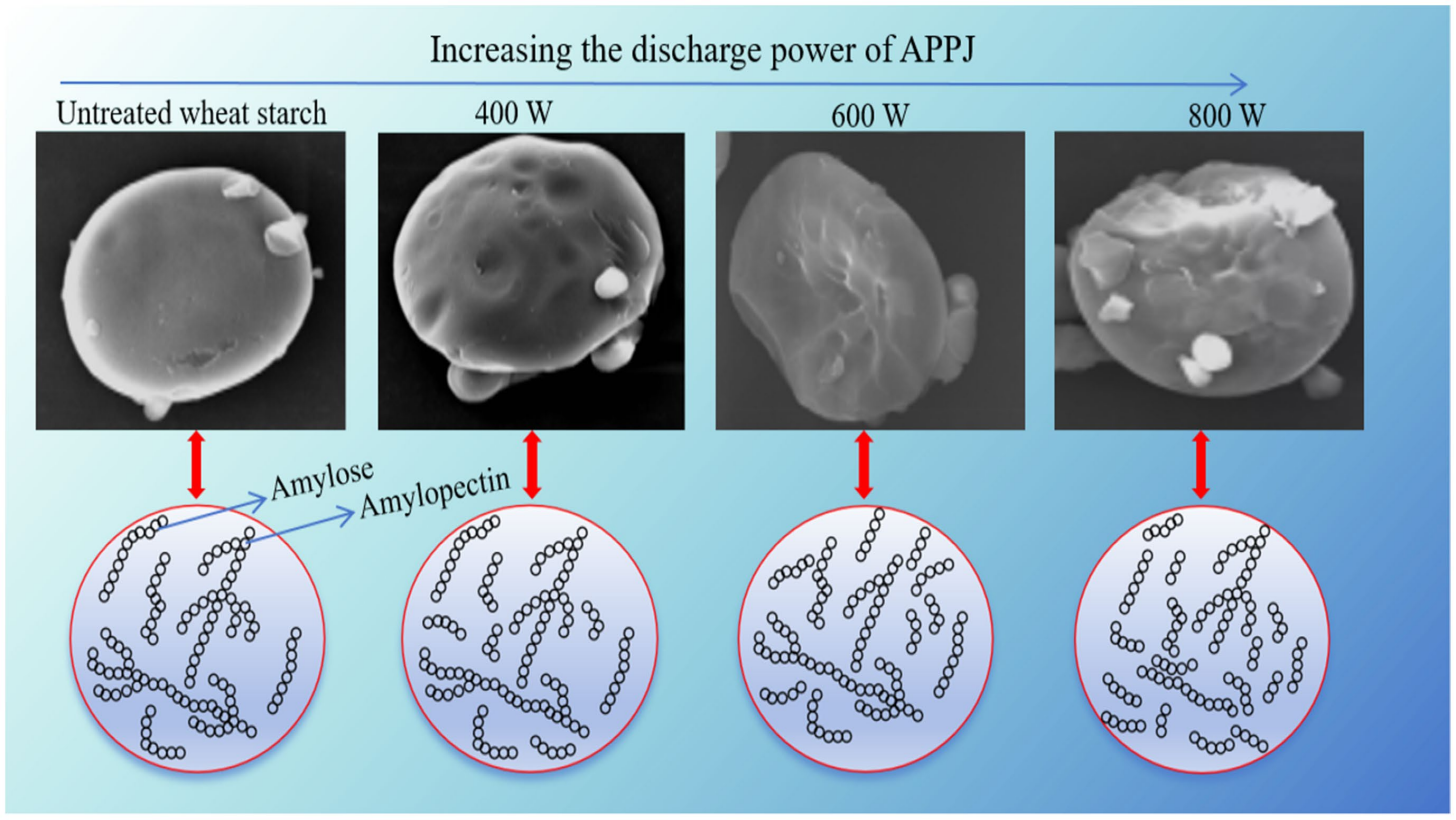
Schematic diagram of the proposed mechanism of the effect of APPJ on wheat starch.

Figure 9 showed schematic diagram of the proposed mechanism of the effect of APPJ on wheat starch. The interaction between APPJ and wheat starch-water suspension could produce reactive oxygen and nitrogen species, such as atomic oxygen, ozone, hydroxyl radicals, hydrogen peroxide, nitrates, nitric oxide, nitrites, and peroxynitrites (42). These active species could etch the surface of wheat starch granules, causing different degrees of fissures, cavities, and fragmentation on the surface of the starch granules (Figures 8, 9). During APPJ treatment, starch chain was decomposed or depolymerized (Figure 9), resulting in the increase of amylose content (Figure 4), the decline of the relative crystallinity (Figure 6), and the dropping of the swelling ability of wheat starch granules (Table 2). The larger the APPJ discharge power, the stronger etching intensity of starch granule and the higher depolymerization rate of starch molecules (Figure 9).

## 4 Conclusion

Wheat starch was subjected to APPJ at different intensities (0, 400, 600, and 800 W, respectively). APPJ obviously changed the surface morphology of wheat starch granules. APPJ treated wheat starch had smaller viscosity such as PV, FV, and BD, and the G′ and G″ of gel was lower than those of the native wheat, too. Notably, APPJ led to a reduction of swelling power and an increase in solubility of starch. However, no significant changes were found in wheat starch’s crystalline structure and FT-IR spectra pattern after APPJ treatments. Therefore, APPJ had the potential to regulate the starch structure and functional properties, especially to enhance the solubility and decrease the viscosity of wheat starch. Future studies should be carried out to explore the effect of APPJ on fine structural parameters and digestive properties of wheat starch under different discharge power and constant effective time.

## Data availability statement

The original contributions presented in the study are included in the article/supplementary material, further inquiries can be directed to the corresponding author.

## Author contributions

HJ: Conceptualization, Data curation, Formal Analysis, Funding acquisition, Investigation, Methodology, Project administration, Resources, Software, Supervision, Validation, Visualization, Writing – original draft, Writing – review & editing. DL: Validation, Visualization, Writing – original draft, Writing – review & editing. LZ: Formal Analysis, Methodology, Software, Supervision, Writing – original draft, Writing – review & editing. ML: Data curation, Writing – original draft, Writing – review & editing. HM: Formal Analysis, Funding acquisition, Resources, Writing – original draft, Writing – review & editing.
